# Value of soluble fms-like tyrosine kinase 1 for predicting acute pancreatitis severity: a systematic review and meta-analysis

**DOI:** 10.1590/1806-9282.20231694

**Published:** 2024-05-20

**Authors:** Sheng-Nan Wang, Tao Tang, Wei-Mei Zhou

**Affiliations:** 1 Zhejiang University, Graduate School of Medical College – Hangzhou, China.; 2 Affiliated Central Hospital of Huzhou University, Huzhou Central Hospital, Department of Gastroenterology – Huzhou, China.

**Keywords:** Vascular endothelial growth factor receptor-1, Acute pancreatitis, Systematic review, Meta-analysis

## Abstract

**OBJECTIVE:**

The objective of this study was to explore the relationship between serum soluble fms-like tyrosine kinase 1 and the severity of acute pancreatitis and its diagnostic utility.

**METHODS:**

This study was carried out by searching Chinese and English literature from the establishment of the database to July 9, 2023, systematically, and assessing the quality and heterogeneity of the articles included.

**RESULTS:**

Thirteen studies with a total of 986 patients were included. Patients with severe acute pancreatitis showed higher levels of soluble fms-like tyrosine kinase 1 compared with mild acute pancreatitis [weighted mean difference=76.64 pg/mL, 95% confidence interval (95%CI 50.39–102.89, p<0.001)]. Soluble fms-like tyrosine kinase 1 predicted pooled sensitivity, specificity, and area under the curve were 79%, 74%, and 0.85 for severe acute pancreatitis, with some heterogeneity (I^2^>50% or p<0.05). In the subgroup analysis, cutoff >150 pg/mL was found to be a heterogeneous factor.

**CONCLUSION:**

Soluble fms-like tyrosine kinase 1 is a reliable tool for identifying acute pancreatitis severity, but only as a screening tool.

## INTRODUCTION

Acute pancreatitis (AP) is an inflammatory disease of varying severity. The majority of patients experience mild acute pancreatitis (MAP), which resolves on its own. However, as per the 2012 Revised Atlanta classification, approximately 30% of patients are categorized as moderately severe acute pancreatitis (MSAP), while around 10% are classified as severe acute pancreatitis (SAP), with some cases dying from AP^1–3^. The early pathological events of AP, whether systemic or local, are associated with endothelial activation and damage^4^. Consequently, persistent endothelial dysfunction and activation are early predictors of the likely severity of AP^5,6^.

Soluble fms-like tyrosine kinase 1 (sFlt-1), also known as vascular endothelial growth factor (VEGF) receptor-1 (VEGFR-1), generated by alternative splicing of Flt-1 pre-mRNA, acts as a membrane receptor that binds VEGF and placental growth factor (PIGF). VEGF is a potent stimulator of endothelial activation and injury^4,6,7^. In AP patients, sFlt-1 was found to be an early marker of the severity of AP patients^6,8^. However, the relevant literature is still vague and needs further clarification and organization.

This meta-analysis aimed to assess the predictive capability of sFlt-1 in determining the severity of AP.

## METHODS

### Search strategy and study identification

A systematic search of publications up to July 9, 2023, in the Cochrane Library, PubMed, EMBASE, China Academic Journals Full-text Database, WANFANG, China Biology Medicine disc, and WEIPU databases was performed to evaluate serum sFlt-1 value as a marker of AP severity. We searched for "pancreatitis" and "Vascular Endothelial Growth Factor Receptor-1" based on "MeSH." We have no restrictions on language or date.

Our inclusion criteria were as follows: (1) a retrospective or prospective study; (2) age >18 years; and (3) serum sFlt-1 can diagnose AP severity that includes MSAP+SAP or SAP (the AP severity was defined by the authors of primary studies). Exclusion criteria were as follows: (1) case reports, reviews, editorials, and animal or in vitro studies; (2) studies without any form of severity stratification; (3) only the abstract was available; (4) duplicate study; and (5) inability to provide complete data required for analysis.

### Data extraction and quality assessment

Three researchers (Wang SN, Tang T, and Zhou WM) selected documents by reviewing abstracts and titles. Data from each study were compiled as follows: first author (year of publication), number of subjects (male/female), study design, age, time of blood sample, sFlt-1 measurement method, mean (SD) and cutoff value of sFlt-1, and sensitivity and specificity of the study. Methodological quality was evaluated independently by two reviewers (Wang SN and Zhou WM) using the QUADAS-2 by RevMan 5.3 (Cochrane Collaboration, Oxford, UK)^9^. All disagreements were settled by consensus among authors.

### Statistical analysis

Systematic reviews and meta-analyses were performed based on the PRISMA checklist^10^. I^2^ and p-values were used to evaluate heterogeneity. When I^2^>50% or p<0.05, the random effects model was used for calculation and analysis, and subgroup analysis was performed to find the source of heterogeneity. The weighted mean difference (WMD) and 95% confidence interval (95%CI) of the included studies were calculated to evaluate the differences in sFlt-1 concentrations between different severity levels. If concentrations were reported as median and IQR in included studies, the corresponding mean and standard deviation were estimated and included in the analysis^11,12^. The pooled sensitivity, specificity, and area under the curve (AUC) of the included studies were calculated. Sensitivity analysis was performed to verify the stability of the results. Publication bias was detected using Deek's funnel plot or Egger's test. All statistical analyses were performed using Stata V.14.0 (Stata Corporation, College Station, TX, USA).

## RESULTS

### Research characteristics

From the initial literature search, we identified and screened 260 references. Of these, 62 were excluded due to duplication. By reading the titles and abstracts, we further removed 181 articles that did not meet the requirements. Among the 17 articles obtained, after reading the full text, five articles were excluded due to failure to obtain complete data, duplicate data, or questionable data. Among them, Dumnicka et al.^5^ reported the results on the first and second days after onset, which were regarded as two articles of this study. Finally, 13 studies were included in the paper^5,8,13–22^. Quality assessment was performed according to QUADAS-2.

### Soluble fms-like tyrosine kinase 1 concentration correlates with acute pancreatitis severity

**Figure 1:**
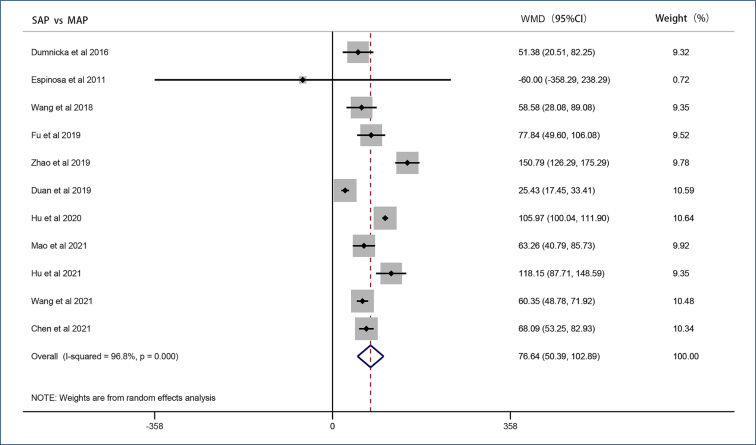
Forest plot of soluble fms-like tyrosine kinase 1 concentrations.

Random-effects results from 11 studies showed that patients with SAP showed higher levels of sFlt-1 compared with MAP (WMD=76.64 pg/mL, 95%CI 50.39–102.89, p<0.001) (Figure 1). In addition, 10 studies showed that patients with AP had higher sFlt-1 levels compared with non-AP patients or healthy adults (WMD=74.80 pg/mL, 95%CI 36.40–113.19, p<0.001). Three studies reported sFlt-1 with a poor prognosis like multiple organ dysfunction and death, and we included them in the poor prognosis group. Results from the fixed-effect model showed significantly elevated sFlt-1 levels in patients with a poor prognosis (WMD=102.76 pg/mL, 95%CI 94.86–110.65, p=0.452).

Except for prognosis groups, significant heterogeneity existed in other groups. Sensitivity analysis showed that excluding any study in the SAP vs. MAP groups and AP vs. non-AP groups, the results did not change significantly. In the prognosis groups, the results changed significantly after excluding the studies of Hu et al. and Mao et al. No significant publication bias was found.

### Diagnostic value of soluble fms-like tyrosine kinase 1 in acute pancreatitis

**Figure 2:**
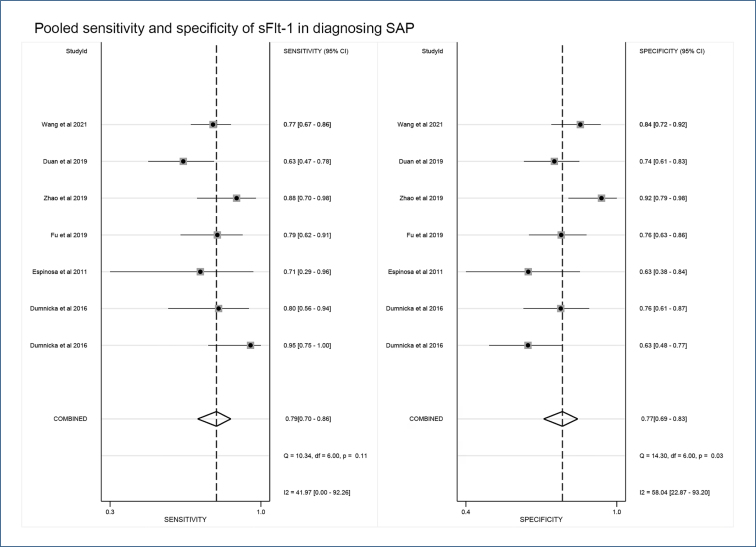
Pooled sensitivity and specificity of soluble fms-like tyrosine kinase 1 in diagnosing severe acute pancreatitis.

Among the included studies, five studies explored the diagnostic value of sFlt-1 for early AP and seven explored the diagnostic value of sFlt-1 for SAP. We performed separate diagnostic meta-analyses. The results showed that the pooled sensitivity, specificity, and AUC of sFlt-1 in predicting AP were 0.78 (95%CI 0.65–0.86), 0.74 (95%CI 0.65–0.81), 0.81 (95%CI 0.77–0.84), I^2^>50%, p<0.05; and in predicting SAP, they were 0.79 (95%CI 0.70–0.86), 0.74 (95%CI 0.69–0.83), 0.85 (95%CI 0.81–0.88), I^2^>40%, p<0.05 (Figure 2). In sensitivity analyses, the deletion of Hu and Wang's studies had a significant impact on the results.

To explore the influencing factors leading to heterogeneity, we performed a subgroup analysis (Table 1). Combined with the overall p-value of the subgroup, the subgroup analysis results of the diagnostic value of sFlt-1 for SAP showed that the "cutoff value (150 pg/mL)" may be an influencing factor of heterogeneity, while no significant influencing factors of heterogeneity were found in the analysis of AP.

**Table 1:** The result of meta-regression and subgroup analysis.

		Category	Parameter	No. of studies	Sensitivity (95%)	P1	Specificity (95%)	P2	p-value	I^2^
SAP	Definition of severity	Yes	Atlanta classification	3	0.85 [0.74–0.96]	0.51	0.68 [0.57–0.79]	0.00	0.04	70
		No	Others	4	0.77 [0.69–0.85]		0.81 [0.75–0.88]			
	Study design	Yes	Prospective	2	0.88 [0.77–0.99]	0.93	0.70 [0.57–0.82]	0.00	0.07	62
		NO	Retrospective	5	0.76 [0.68–0.83]		0.79 [0.73–0.86]			
	Geographical location	Yes	Asia	4	0.77 [0.69–0.85]	0.02	0.81 [0.75–0.88]	0.41	0.04	70
		No	Others	3	0.85 [0.74–0.96]		0.68 [0.57–0.79]			
	sFlt-1 sampling time	Yes	≤24 h	2	0.82 [0.75–0.89]	0.55	0.77 [0.70–0.85]	0.13	0.17	44
		NO	>24 h	2	0.69 [0.57–0.81]		0.75 [0.63–0.87]			
	Sample size	Yes	>100	2	0.72 [0.63–0.82]	0.00	0.79 [0.68–0.90]	0.15	0.10	57
		NO	≤ 100	5	0.84 [0.77–0.91]		0.75 [0.67–0.84]			
	Cutoff value	Yes	>150 pg/mL	3	0.81 [0.70–0.92]	0.39	0.83 [0.76–0.90]	0.21	0.00	84
		NO	≤150 pg/mL	3	0.78 [0.65–0.91]		0.71 [0.64–0.78]			
	Methods	Yes	ELISA	5	0.76 [0.68–0.83]	0.02	0.79 [0.73–0.86]	0.37	0.07	62
		NO	ECLIA	2	0.88 [0.77–0.99]		0.70 [0.57–0.82]			
AP	Definition of severity	Yes	Atlanta classification	1	0.81 [0.60–1.00]	0.99	0.62 [0.53–0.71]	0.00	0.04	68
		No	Others	4	0.76 [0.64–0.88]		0.79 [0.72–0.85]			
	Study design	Yes	Prospective	2	0.84 [0.73–0.95]	0.97	0.77 [0.64–0.89]	0.31	0.28	22
		NO	Retrospective	3	0.72 [0.58–0.86]		0.72 [0.62–0.82]			
	sFlt-1 sampling time	Yes	≤24 h	3	0.81 [0.70–0.92]	0.89	0.71 [0.61–0.81]	0.03	0.55	0
		NO	>24 h	2	0.71 [0.54–0.89]		0.78 [0.67–0.88]			
	Sample size	Yes	>100	1	0.51 [0.42–0.61]	0.00	0.79 [0.65–0.92]	0.48	0.00	82
		NO	≤100	4	0.82 [0.78–0.87]		0.73 [0.63–0.82]			
	Cutoff value	Yes	>150 pg/mL	3	0.83 [0.74–0.92]	0.86	0.68 [0.59–0.77]	0.00	0.05	66
		NO	≤150 pg/mL	2	0.66 [0.51–0.82]		0.81 [0.72–0.90]			

The funnel plot of the diagnostic value of sFlt-1 for AP found significant bias, mainly from the study published by Duan, while SAP diagnostic value funnel plots did not show publication bias.

## DISCUSSION

In this study, we determined that sFlt-1 is related to AP severity and is a moderate predictor of AP severity, with AUC, sensitivity, and specificity of 0.85, 79, and 74%, respectively. However, due to the small number of studies, there is no significant correlation between elevated sFlt-1 and the primary outcome of AP patients, and its value in assessing the prognosis of AP needs to be further explored. The above results indicate that sFlt-1 has a high false negative rate and false positive rate. Therefore, the use of sFlt-1 cannot be the gold standard for AP severity.

Soluble fms-like tyrosine kinase 1 is the soluble isoform of Flt-1 and has extremely high affinity with VEGF and PIGF, which will bind to them and hinder their angiogenic effects on VEGFR, and is considered a marker of endothelial dysfunction^14,23^. The early pathological events of AP are related to vascular disorders^4^, involving the activation and dysfunction of endothelial cells^24^. Studies have found that VEGF is expressed increasingly in the inflamed pancreas, which is consistent with increased vascular permeability in early pancreatitis^4^. In some inflammatory diseases, circulating sFlt-1 levels are elevated and positively correlated with severity, suggesting adverse clinical events^25–27^. In summary, endothelial injury may also cause an increase in sFlt-1 in AP, which may reflect the severity of AP or organ dysfunction. This was confirmed in our meta-analysis.

As with any other laboratory marker, the accuracy of sFlt-1 appears to be dependent on cutoff values^28^. When the sFlt-1 value is >150 pg/mL, there is no difference in sensitivity and specificity between groups, but there is significant heterogeneity in the summary results (p<0.01), which may be the source of study heterogeneity. As we selected the same cutoff value for subgroup analysis, sFlt-1 was not found to be a source of heterogeneity in the analysis of the diagnostic value of sFlt-1 for AP. Therefore, we believe that for SAP, a higher cutoff value may be needed. Of course, the optimal cutoff value of sFlt-1 for both AP and SAP still needs to be discussed in future studies.

The expression of sFlt-1 is closely related to the timing of AP severity. There was no heterogeneity regardless of sampling time (p=0.19). Nearly every study measured sFlt-1 within 48 h of onset. However, when the sFlt-1 assay measured within 24 h was used to predict AP severity, increased sensitivity (82 vs. 69%) and similar specificity (77 vs. 75%) were found. Kolber et al. reported 95 AP patients admitted within 24 h of onset^29^. The sFlt-1 values in SAP and MSAP on the second day decreased compared with the first day. Likewise, Dumnicka et al. found that sFlt-1 predicted an AUC of 0.808 for MSAP+SAP in 66 patients. However, subsequent subgroup analysis of the onset time from 18 to 21 h found that the AUC increased (0.836)^5^. In addition, the AUC dropped to 0.791 on the second day of admission. The above results suggest that the peak expression of sFlt-1 may be 24 h after onset, but the specific time period is currently unclear and needs further research and exploration.

There are several other limitations to this study. First, heterogeneity was large in some analyses. Although the random effects model, sensitivity analysis, and subgroup analysis were used to correct the results and find sources of heterogeneity, the heterogeneity could not be completely removed. Second, the included articles are all in English or Chinese, and most of them are Chinese articles. Third, the dynamic nature of organ failure has been found to be critical for AP in recent years, as persistent organ failure carries a worse prognosis than transient organ failure^1,30^. Unfortunately, there are no original articles containing data on persistent organ failure, making it impossible to discuss this issue. Finally, the difference in cutoff values is observed among studies. Hence, we did not get a certain cutoff value, which could lead to the clinical application of the sFlt-1 being limited.

## CONCLUSION

Serum sFlt-1 can only be used as a screening tool for the severity of AP. However, studies focusing on different determinations of sFlt-1, the timing of sFlt-1 measurement, and the consistency of definitions of AP severity may improve the accuracy of sFlt-1 in estimating AP severity.
