# ERRATUM: Value of soluble fms-like tyrosine kinase 1 for predicting acute pancreatitis severity: a systematic review and meta-analysis

**DOI:** 10.1590/1806-9282.20231694-ER

**Published:** 2026-01-09

In the manuscript: Wang S-N, Tang T, Zhou W-M. Value of soluble fms-like tyrosine kinase 1 for predicting acute pancreatitis severity: a systematic review and meta-analysis. Rev Assoc Med Bras. 2024;70(5):e20231694. https://doi.org/10.1590/1806-9282.20231694

Where it reads:

^1^Zhejiang University, Graduate School of Medical College – Hangzhou, China.

It should read:

^1^Huzhou Hospital of Zhejiang University School of Medicine, Huzhou Central Hospital – Huzhou, China.

Roseli Nomura

Editor-in-Chief

